# Comparison of the Effect of Anesthetic Agents on Blood Levels of Parathyroid Hormone and Ionized Calcium: A Prospective Randomized Controlled Trial

**DOI:** 10.1155/2022/7795004

**Published:** 2022-05-18

**Authors:** Esra Mercanoglu Efe, Turkay Kirdak, Gulnihal Aykut, Elif Kaya Argadal, Ekrem Kaya, Canan Ersoy

**Affiliations:** ^1^Uludag University Medical Faculty, Department of Anesthesiology and Reanimation, Bursa, Turkey; ^2^Uludag University Medical Faculty, Department of General Surgery, Bursa, Turkey; ^3^Uludag University Medical Faculty, Department of Biochemistry, Bursa, Turkey

## Abstract

The aim of this randomized control trial is to compare the effect of anesthetic agents on blood levels of parathyroid hormone and ionized calcium. 77 American Society of Anesthesiologists I-II patients who would undergo laparoscopic cholecystectomy were enrolled into this prospective study and randomized into 3 groups with sealed envelope technique as Group S: sevoflurane, Group D: desflurane, and Group TIVA: total intravenous anesthesia. The first blood sample was used to check the baseline blood levels of parathyroid hormone and ionized calcium. In Group S or D, maintenance of anesthesia was being performed with 1 MAC (minimum alveolar concentration) sevoflurane or desflurane, respectively, while in Group TIVA, it was performed with 150 mcg/kg/min propofol and 1 mcg/kg/min remifentanil IV infusions. At the 30^th^ minute of anesthesia and at the 1^st^ hour of end of anesthesia, 2^nd^ and 3^rd^ blood samples, respectively, were used to check the blood levels of PTH and Ca. During perioperative period, hemodynamic parameters were also noted. Blood levels of parathyroid hormone at the 30^th^ min after anesthesia were found to be significantly different between groups (*P*=0,01). The PTH level at the 30^th^ min after anesthesia was found significantly higher in Group S than that of Groups D and TIVA (*P*=0.005 and *P*=0.001, respectively). Blood levels of ionized calcium at 30^th^ min after anesthesia were found significantly different between groups (*P*=0,048). It was found significantly higher in Group TIVA than that in Group S (*P*=0.024). Desflurane seems to be the best agent for parathyroidectomy procedures. Future research studies are needed to be conducted to reach out more correct and valuable outcomes.

## 1. Introduction

Thyroidectomy and parathyroidectomy are common surgeries in which general anesthesia has to be administered [1–3]. These cases are needed to be monitored closely for parathyroid hormone (PTH) and ionized calcium (Ca) levels which have an unstable statement. Lower levels of PTH can lead to an important decrease in blood Ca levels [3–6]. This low blood level of Ca causes aches, cramping, twitching of muscles or tetany (involuntary muscle contraction), fatigue, weakness, depression, or anxiety [3, 4, 6]. During cervical area surgeries, PTH levels may be affected by several factors [7]. Most often, accidental surgical resection of the parathyroid tissues can cause hypoparathyroidism [3–5]. The aim of this prospective randomized controlled study was to find out and compare the effect of different anesthetic agents on blood levels of PTH and Ca during the perioperative period of laparoscopic cholecystectomy procedures.

## 2. Material and Methods

After obtaining approval of the consent study from the institutional ethics committee (number: 2014–21/10, date: 11/Nov/2014) and written informed consent from participants, this prospective, randomized-designed study was carried out at general surgery operating room, Uludag University Medical Faculty, Bursa, Turkey. 77 American Society of Anesthesiologists (ASA) I-II patients, who were scheduled for laparoscopic cholecystectomy, were enrolled into the study.

While ASA I-II patients were being included in the study, exclusion criteria were refusal to join the study, history of difficult intubation or predicted difficult airway, chronic osteoporosis and renal failure, transfusion of any blood products and previous antibiotics treatment during the last one month, diuretics treatment, and history of thyroidectomy or parathyroidectomy operations. Also, some patients whose baseline blood levels of PTH and Ca were abnormal and operations were turned to open cholecystectomy, who had unanticipated difficult intubation were excluded from the study after being included.

Randomization was performed into 3 groups with the sealed envelope technique as Group S: sevoflurane, Group D: desflurane, and Group TIVA: total intravenous anesthesia. The patient's name, protocol, age, gender, height, weight, body mass index, comorbidities, medicines, previous operations, smoke and alcohol intake, and any allergies were recorded before entering the operating room. The patients were not premedicated. In the operating room, noninvasive blood pressure, peripheral oxygen saturation (SpO2), and electrocardiography (ECG) were monitored and a peripheral intravenous line was inserted to infuse 0.9% NaCl. The first blood sample was taken from a separate intravenous line just before anesthesia to check the baseline blood levels of PTH and Ca. This line was used only for blood samples. Routine general anesthesia was induced with 0.03 mg/kg midazolam, 2 mg/kg propofol, and 2 mcg/kg fentanyl. For neuromuscular blockade, 0.6 mg/ideal weight kg rocuronium was administered. After providing suitable conditions, the patient was intubated and started to be ventilated with a 2 lt flow of 50 : 50 O_2_ and an air mixture with PEEP (positive end-expiratory pressure) of 5 cmH_2_O to adjust EtCO_2_ (end-tidal carbon dioxide) around 35 mmHg. An oral gastric tube was inserted for the intraoperative period. During this laparoscopic procedure, CO_2_ (carbon dioxide) was used for gas insufflation and the intra-abdominal pressure was maintained between 12 and 14 mmHg during the intraoperative period.

Maintenance of general anesthesia was performed according to the study group. In Group S or D, it was being performed with 1 MAC (minimal alveolar concentration) sevoflurane or desflurane, while in Group TIVA, it was performed with 150 mcg/kg/min propofol and 1 mcg/kg/min remifentanil infusions. At the 30^th^ minute of anesthesia and at the 1^st^ hour of the end of anesthesia, 2^nd^ and 3^rd^ blood samples, respectively, were obtained to check the blood levels of PTH and Ca. Fifteen minutes before the end of the surgery, tenoxicam and paracetamol IV (intravenous) were administered for postoperative pain management and metoclopramide IV was administered for antiemetic prophylaxis. During the perioperative period (before anesthesia induction (BAI), after intubation (AI), at 30^th^ min of anesthesia, at the end of the operation, and at 1^st^ hour of end of anesthesia), hemodynamic parameters such as heart rate (HR), systolic arterial pressure (SAP), diastolic arterial pressure (DAP), mean arterial pressure (MAP), SpO_2_, and EtCO_2_ were also recorded. SAP more than 20% or less than 20% of the beginning SAP was considered as hypertension and hypotension, respectively, and both were managed with proper position and medication. After the end of the surgery, all patients were extubated and sent to the recovery room and the clinics in order.

The accordance of the data to normal distribution was examined with the Shapiro–Wilk test and Kolmogorov–Smirnov test. For descriptive statistics of continuous variables, the standard deviation was used for those which were conforming to the distribution average and for those which were not conforming to the normal distribution median (minimum-maximum) was used. The descriptive statistics for categorical variables were given as percentile (*n*%). For comparing the independent groups of continuous variables, those which were conforming to the normal distribution were analyzed with one-way variance analysis (one-way ANOVA) and those which were not conforming to the normal distribution were analyzed with Kruskal–Wallis H test. When a significance was found after Kruskal–Wallis test, the dual comparisons of the groups were analyzed with the Mann–Whitney *U* test. For comparisons of the categorical variables between the groups, Pearson's chi-squared test and Fisher–Freeman–Halton tests were used. For the explanation of the change according to the initial value of the time-dependent variables, the comparison of the group's percent variable value ((last value−first value)/first value) was used. SPSS 26.0 software program was used for the analysis of the study. The significance level was accepted at *α* = 0.05.

## 3. Results

Demographic variables were not found statistically different between groups (Table 1). Also, hemodynamic variables (Table 2) and baseline blood levels of PTH and Ca were not found to be statistically different between groups. PTH blood levels at the 30^th^ min after anesthesia was found to be significantly different between groups (*P*=0,01). While there were no differences between Groups D and TIVA, the PTH level at the 30^th^ min after anesthesia was found to be significantly higher in Group S than that in Groups D and TIVA (*P*=0.005 and *P*=0.001, respectively) (Table 3). Ca blood levels at the 30^th^ min after anesthesia was found significantly different between groups (*P*=0,048). While there were no differences between Groups S and D, also between D and TIVA, it was found significantly higher in Group TIVA than that in Group S (*P*=0.024) (Table 4). Blood levels of PTH and Ca at other times were found to be statistically similar between groups.

## 4. Discussion

Using an ideal anesthetic agent is still confusing for anesthesiologists. Although there are many numbers of surgical reviews for choosing the best surgical techniques in parathyroidectomy cases [7], anesthesiologists still do not know the best anesthetic technique for parathyroidectomy procedures.

According to this present study, in Group D and Group TIVA, the PTH levels at the 30^th^ minute were similar. However, this value of Group S was significantly higher than that of Group D and Group TIVA. Also, Ca levels at the 30^th^ minute were significantly higher in Group TIVA than those in Group S. So, desflurane seems to be the best agent for parathyroidectomy or thyroidectomy cases.

This research is very valuable and unique because it is the first prospective randomized study that compares blood levels of PTH and Ca between different anesthetic techniques during laparoscopic cholecystectomy procedures in humans. That is why we cannot discuss or compare our data with other similar ones.

There is a lot of research that compares blood levels of PTH and Ca in parathyroidectomy cases [7–9]. Surgical interventions to parathyroid glands and the cervical area, such as thyroidectomy and parathyroidectomy procedures, may cause a fluctuation in hormonal levels [9, 10]. Our research was planned for only laparoscopic cholecystectomy cases due to being apart from the parathyroid gland and cervical areas. Therefore, we believed that our results would not have been affected by this hormonal fluctuation and would have been more reliable and independent.

Chonkich et al. found that low blood levels of ionized Ca and the associated increase in PTH and osteocalcin following isoflurane anesthesia were due to a combination of factors. Also, they reported that additional experiments were needed to identify the mechanisms that control the uptake of Ca by bone or soft tissue in the presence of isoflurane [1]. In our study, sevoflurane was found to be more likely to increase blood levels of PTH at the 30^th^ minute compared to desflurane and TIVA. However, blood levels of Ca levels were found higher in the TIVA group according to the sevoflurane group. Actually, if Ca levels were just dependent on PTH, they would have been higher in the sevoflurane group. But some previous reviews explain it as the propofol's effect on PTH levels by increasing catecholamines as a result of respiratory acidosis after propofol administration [7]. Also, they advise more studies including rapid sequential sampling for more than 9 minutes postinduction [7]. Since we did not consider catecholamine release which affects blood levels of PTH, our blood samples were taken at the 30^th^ minute of the postinduction period. Also, we monitored end-tidal CO_2_ levels in all cases and avoided respiratory acidosis. Also, we believe that catecholamine releases are dependent on lots of other self conditions, like current systemic and even physicological diseases. Some ASA II cases were using drugs which would affect catecholamine levels. So, the catecholamine level was not a suitable variable that could be taken into account.

Hong et al. found samples of preincision intraoperative blood levels of PTH (IOPTH) should be drawn before induction to avoid potentially inappropriate incorporation of anesthetic-related elevations in IOPTH into values used for surgical decision and they analyzed transient increases in IOPTH related to anesthetic technique [7]. In our study, preincision IOPTH was analyzed too and the operational side was away from parathyroid glands as an advantage over the studies that compared PTH levels in parathyroidectomy cases.

In previous studies, propofol was found not to interfere with PTH assays at clinically relevant concentrations. There is no evidence to support the avoidance of a propofol anesthetic for parathyroid surgery [11]. In our study, desflurane was found to be the least effective agent on blood levels of PTH and Ca.

Kivela et al. found that PTH concentrations in patients with primary hyperparathyroidism were not affected by the type of anesthesia (propofol *vs* sevoflurane) [11]. In our research, the PTH level at the 30^th^ min after anesthesia was found to be significantly higher in Group S according to Group D and Group TIVA. Again, Kivela et al. found that intravenous propofol infusion did not alter PTH levels significantly during the operation. They believed the intraoperative PTH assay could be used safely during propofol sedation during parathyroid surgical procedures [11].

Respiratory alkalosis can precipitate hypocalcemia by decreasing blood levels of ionized calcium [10]. In our study, we considered staying in standard normalized range of EtCO_2_ during ventilating in all intubated cases. Unfortunately, we did not analyze arterial blood gases during operations. This might be one of the limitations of our study and could be searched in the future studies.

In a research study, propofol with or without EDTA (ethylenediaminetetraacetic acid) was used for sedation in healthy cases, and the researchers found that propofol without EDTA increased PTH levels in normal subjects; however, propofol with EDTA did not alter blood ionized calcium or total magnesium concentrations [12]. In our study, we use propofol infusion for our TIVA group. In Group S (sevoflurane), blood levels of PTH at the 30^th^ min of operation were found to be higher according to the Group TIVA.

Also, in a study that was aimed to determine the effect of propofol on intraoperative blood levels of PTH testing, the researchers performed a clinical trial on patients with secondary hyperparathyroidism who underwent a nonparathyroid operation. In this trial, they randomly assigned patients to receive propofol or nonpropofol anesthesia and tested PTH levels during the operation [13]. They found that there were no statistical differences between the two groups on PTH levels. This study was similar to ours about working on nonparathyroid operations but our patients' PTH levels were normal in the first blood samples.

As mentioned before in our study, we worked in a noncervical surgical area, so we decided to choose laparoscopic cholecystectomy which was less stressful operation for patients' metabolism because the duration was short and generally, the age of cases was average. However, surgeons and anesthetists were not the same, so the stress of operations and intubations could be different. This might be another limitation of our study.

Most of the research studies and we also concluded that future randomized clinical trials are needed to arrive at a consensus for safe parathyroidectomy cases.

As a result of this present study, according to activity on blood levels of PTH for the intraoperative period, desflurane and TIVA are being seemed to be less effective agents than sevoflurane, and according to activity on blood levels of Ca for the intraoperative period, desflurane and sevoflurane seem to be less effective agents than TIVA. So, because desflurane is the common agent that affects both blood levels PTH and Ca less than the others, it seems to be the best agent for parathyroidectomy and thyroidectomy procedures according to our research.

Moreover, we believe that future studies about the effect of more anesthetic agents on perioperative blood levels of PTH and Ca and also other elements, during different kinds of operational procedures apart from parathyroid glands, with the same surgeon, same anesthetist, and with arterial blood gases analyses are needed to be performed to reach out more correct, valuable, and reliable outcomes.

## Figures and Tables

**Table 1:** Demographic variables between groups.

	Group S (*n* = 25)	Group D (*n* = 26)	Group TIVA (*n* = 26)	*P* value
ASA				0.766^a^
ASA 1	12 (48)	10 (38.5)	12 (46.2)	
ASA 2	13 (52)	16 (61.5)	14 (53.8)	
Gender				0.228^a^
F	19 (76)	18 (69.2)	14 (53.8)	
M	6 (24)	8 (30.8)	12 (46.2)	
Age (yr)^*∗*^	49 (18 : 75)	44 (28 : 86)	48 (20 : 74)	0.697^b^
BMI^*∗*^	28.76 (15.94 : 40.16)	27.26 (21.36 : 52.07)	28.22 (21.48 : 49.82)	0.989^b^
Weight (kg)^*∗*^	72 (45 : 118)	79 (61 : 130)	81 (55 : 134)	0.484^b^
Smoke				0.585^a^
+	6 (24)	9 (34.6)	6 (23.1)	
−	19 (76)	17 (65.4)	20 (76.9)	
Alcohol				0.759^c^
+	2 (8)	4 (15.4)	4 (15.4)	
−	23 (92)	22 (84.6)	22 (84.6)	
Allergy				0.759^c^
+	3 (12)	4 (15.4)	2 (7.7)	
−	22 (88)	22 (84.6)	24 (92.3)	
Duration of anesthesia (min)^*∗∗*^	67.44 ± 12.14	70.88 ± 18.07	72.12 ± 20.55	0.611^d^
Duration of surgery (min)^*∗*^	60 (35 : 85)	60 (30 : 110)	61 (20 : 100)	0.429^b^

Values were represented as mean ± standard deviation, median (minimum : maximum) and *n* (%). ^*∗*^Median (min : max); ^*∗∗*^Mean ± SD. F: female; M: male; yr: year; BMI: body mass index; min: minute; ^a^Pearson chi-square test; ^b^Kruskal–Wallis H test; ^c^Fisher–Fremann Halton test; ^d^One-way ANOVA. Groups are homogeneous in terms of demographic variables.

**Table 2:** Hemodynamic parameters between groups.

	Group S (*n* = 25)	Group D (*n* = 26)	Group TIVA (*n* = 26)	*P* value
HR^*∗*^	−0.03 (−0.30 : 1.22)	0.04 (−0.11 : 0.74)	0.01 (−0.24 : 0.33)	0.215^a^
SBP^*∗∗*^	−0.17 ± 0.17	−0.13 ± 0.13	−0.11 ± 0.17	0.289^b^
DBP^*∗*^	−0.12 (−0.36 : 0.50)	−0.15 (−0.44 : 0.08)	−0.14 (−0.40 : 0.55)	0.701^a^
SpO_2_^*∗*^	0.01 (0 : 0.04)	0.01 (0 : 0.03)	0.01 (0 : 0.05)	0.614^a^
ETCO_2_^*∗*^	0.06 (−0.06 : 0.27)	0.06 (−0.12 : 0.32)	0.05 (−0.03 : 0.23)	0.844^a^

Data were compared by taking percentage change values ((value postintubation−value before intubation)/value before intubation). ETCO_2_ percentage change value = ((value of the 30^th^ min ETCO_2_–value of the 1^st^ min ETCO_2_)/value of the 1^st^ min ETCO_2_. Values were represented as mean ± standard deviation, median (minimum : maximum). ^*∗*^Median (min : max) ^*∗∗*^Mean ± SD. ^a^Kruskal–Wallis H Test; ^b^One-way ANOVA. HR: heart rate; SBP: systolic blood pressure; DBP: diastolic blood pressure; SpO_2_: peripheral oxygen saturation; ETCO_2_: end-tidal carbon dioxide. Groups are homogeneous in terms of hemodynamic parameters.

**Table 3:** Blood levels of PTH and Ca.

	Group S (*n* = 25)	Group D (*n* = 26)	Group TIVA (*n* = 26)	*P* value
Blood levels of Ca at baseline	9 (6.1 : 9.9)	9.1 (8.1 : 9.9)	9.2 (7.6 : 10.2)	0.565^a^
Blood levels of PTH at baseline	51.3 (16.2 : 129.9)	65 (22.2 : 151.8)	57.5 (29.1 : 215.2)	0.668^a^
Blood levels of Ca at the 30^th^ minute	8.4 (4.6 : 9.7)	8.6 (7.3 : 9.8)	8.8 (8 : 9.6)	0.074^a^
Blood levels of PTH at the 30^th^ minute	125.1 (52.5 : 179.1)	102.3 (26.5 : 314.8)	91.4 (45.4 : 354.3)	0.166^a^
Blood levels of PTH at the 30^th^ minute percentage change	1.16 (−0.39 : 10.1)	0.62 (0 : 2.2)	0.60 (−0.25 : 2.03)	0.001^a^
Blood levels of Ca at the 30^th^ minute percentage change	−0.074 (−0.25 : 0.41)	−0.067 (−0.19 : 0.02)	−0.041 (−0.10 : 0.09)	0.048^**a**^

Data were compared by taking percentage change values and written as median (minimum : maximum) ((blood level at the 30th minute–blood level at baseline)/blood level at baseline). Values were represented as median (Minimum-maximum). ^a^Kruskal–Wallis H test.

**Table 4:** Pairwise comparisons between groups.

	Group S-Group D	Group S-Group TIVA	Group D-Group TIVA
Blood levels of PTH at the 30^th^ minute percentage change	0.005^b^	0.001^b^	0.323^b^
Blood levels of Ca at the 30th minute percentage change	0.429^b^	0.024^b^	0.064^b^

Data were compared by taking percentage change values ((blood level at the 30^th^ minute–blood level at baseline)/blood level at baseline). ^b^Mann–Whitney *U* Test.

## Data Availability

Access to the data is restricted.
